# Factors associated with the development of bacterial pneumonia and the preventive potential of peroral endoscopic myotomy in patients with esophageal motility disorders: a case–control study

**DOI:** 10.1007/s00535-025-02238-8

**Published:** 2025-03-24

**Authors:** Hitomi Hori, Hirofumi Abe, Shinwa Tanaka, Hiroya Sakaguchi, Kazunori Tsuda, Chise Ueda, Fumiaki Kawara, Takashi Toyonaga, Masato Kinoshita, Satoshi Urakami, Tatsuya Nakai, Shinya Hoki, Hiroshi Tanabe, Yuzo Kodama

**Affiliations:** 1 Division of Gastroenterology, Department of Internal Medicine, Graduate School of Medicine School of Medicine, Kobe University, 7-5-1 Kusunoki-Cho, Chuo-Ku, Kobe, Hyogo 650-0017 Japan; 2 Department of Endoscopy, Kobe University Hospital, Kobe, Hyogo Japan

**Keywords:** Esophageal motility disorders, Esophageal achalasia, Pneumonia, Aspiration, POEM

## Abstract

**Background:**

Patients with esophageal motility disorders (EMDs) sometimes develop bacterial pneumonia (BP). However, factors associated with BP in patients with EMDs and whether peroral endoscopic myotomy (POEM) reduces BP development are unclear. Therefore, this study aimed to identify factors associated with BP development and evaluate the preventive potential of POEM in patients with EMDs.

**Methods:**

This study included 623 patients diagnosed with EMDs at our institution between April 2015 and March 2023. Factors associated with BP were analyzed by comparing characteristics between patients who developed BP within 1 year before diagnosis using multivariable analysis. The potential of POEM to prevent BP development was assessed using Cox regression analysis, considering treatment status as a time-varying covariate.

**Results:**

Of the 623 patients, 31 (5.0%) developed BP within 1 year before diagnosis. Older age (odds ratio [OR] = 1.29, 95% confidence interval [CI] 1.04–1.59, *p* = 0.019; 10-year increments), lower body mass index (OR = 0.87, 95% CI 0.78–0.98, *p* = 0.026), and manometric diagnosis of spastic esophageal disorders (OR = 2.97, 95% CI 1.24–7.16, *p* = 0.015) were significantly associated with BP. Treatment status of POEM was proved to be a significant factor for developing BP using Cox regression analysis (hazard ratio = 0.17, 95% CI 0.039–0.75, *p* = 0.019).

**Conclusions:**

Risk factors associated with BP in patients with EMDs were older age, lower body mass index, and manometric diagnosis of spastic esophageal disorders. POEM could decrease spasm-related bolus reflux, improve patients’ nutritional status through resolution of transit disturbance, and reduce respiratory complications, suggesting that POEM could help prevent BP development.

**Supplementary Information:**

The online version contains supplementary material available at 10.1007/s00535-025-02238-8.

## Introduction

Esophageal motility disorders (EMDs), such as achalasia and non-achalasia, are characterized by impaired relaxation of the lower esophageal sphincter and abnormal contractions of the esophageal body, leading to dysphagia, food retention, regurgitation, and weight loss [1, 2]. Furthermore, some patients with EMDs develop bacterial pneumonia (BP) due to aspiration [3–6].

BP is pneumonia caused by bacterial pathogens and a leading cause of hospitalization and death in the elderly population. Various risk factors are known to play a role in the development of pneumonia, including decreased immune function of the host and factors that predispose to aspiration, such as old age, dysphagia, dementia, and increased chance of gastric contents reaching the lung (reflux and tube feeding) [7]. Although pneumonia due to aspiration is common in older adults, cases of bacterial pneumonia and pyothorax have been reported in younger patients with EMDs [3–6]. Furthermore, food retention and reflux may increase the risk of developing bacterial pneumonia due to aspiration. However, the risk factors for BP in patients with EMDs remain unclear.

In addition, in recent years, peroral endoscopic myotomy (POEM) has become known and widely performed owing to its safety and clinical effectiveness as treatment of EMDs [8, 9]. POEM significantly improves achalasia-related symptoms and enhances social functioning [10]. Moreover, dysphagia and an increased chance of gastric contents reaching the lung are considered risk factors for the development of BP due to aspiration [7]. Performing POEM may improve dysphagia and food regurgitation; however, whether POEM could be effective in preventing the development of BP in patients with EMDs remains unclear. Therefore, this study aimed to investigate the risk factors for the development of BP in patients with EMDs and determine whether POEM could help prevent BP in patients with EMDs.

## Methods

### Ethics statements

This study adhered to The Strengthening the Reporting of Observational Studies in Epidemiology statement [11]. The opt-out method was used to obtain consent from all study patients. The study protocol was conducted in accordance with the Declaration of Helsinki and approved by the Ethics Committee of Kobe University Hospital (approval number: B220191).

### Study design and setting

This single-center case–control study was conducted at the Kobe University Hospital from April 2023 to March 2024. Patients diagnosed with EMDs between April 2015 and March 2023 were enrolled; clinical data were retrospectively collected from a prospectively maintained institutional database.

### Participants

This study included patients of all ages diagnosed with EMDs by high-resolution manometry (HRM), endoscopy, and esophagography between April 1, 2015, and March 31, 2023. The reason for including all age groups was to accurately analyze the risk factors for developing BP, as there have been case reports of pediatric patients with achalasia developing pneumonia [12]; thus, acknowledging the possibility of many young patients with BP.

### Bacterial pneumonia

BP is pneumonia caused by a bacterial pathogen [13]. However, due to the low identification rate of pathogens in BP cases in previous reports [14], we defined BP in patients with EMDs as pneumonia requiring antibiotic therapy. Patients with a history of pneumonia without detailed information and those with pulmonary infections due to non-tuberculous mycobacteria and *Mycobacterium tuberculosis* were excluded. In addition, pneumonia occurring in patients with a history of interstitial pneumonia was excluded.

### Variables

The Eckardt score consists of four score categories, dysphagia, regurgitation, chest pain, and weight loss, each rated on a 0–3-point scale [15]. The manometric diagnosis was based on the Chicago classification version 3.0 [16], and achalasia was categorized as type I, type II, type III, distal esophageal spasm (DES), jackhammer esophagus, esophagogastric junction outflow obstruction (EGJOO), and others. Furthermore, achalasia type III, DES, and jackhammer esophagus were defined as manometric diagnoses of spastic esophageal disorder [17]. The integrated relaxation pressure (IRP) was measured using the Starlet system (StarMedical, Tokyo, Japan) [16]. The morphology of the esophagus was classified by the angle of inflection into straight (≥ 135°), sigmoid type 1 (≥ 90°, < 135°), and sigmoid type 2 (< 90°). Esophageal dilatation was classified into grade I (d < 3.5 cm), grade II (3.5 cm ≤ d < 6.0 cm), and grade III (d ≥ 6.0 cm) according to the maximum diameter [d] of the barium esophagram.

### Data source

The survey items included the following: age at EMD diagnosis, sex, body mass index (BMI), duration of symptoms, American Society of Anesthesiologists-physical status (ASA-PS), Eastern Cooperative Oncology Group-physical status (ECOG-PS), pretreatment Eckardt score, IRP, Chicago classification by HRM [16], presence of food retention during endoscopy, esophageal morphology, and esophageal dilation. All enrolled patients were followed up during the enrollment period on the basis of the information available in their medical records.

### Peroral endoscopic myotomy

Patients aged 5 years and older, weighing at least 15 kg, with achalasia and non-achalasia diseases amenable to general anesthesia were eligible for POEM treatment. The POEM procedure was performed in four steps with patients under general anesthesia and carbon dioxide insufflation as follows: (i) creation of a mucosal entry, (ii) creation of a submucosal tunnel, (iii) myotomy, and (iv) closure of the entry, following the standard protocol of Inoue et al. [2]. The Flush knife (BTS 3.0; DK-2620 J-B30S; Fujifilm, Tokyo, Japan) was used for all procedures.

### Statistical analysis

Categorical variables are expressed as frequencies with percentages and continuous variables as medians (interquartile ranges [IQRs]). Categorical variables were compared between patients with EMDs who developed BP and those who did not using the Chi-square test or Fisher exact test. Continuous variables were compared using the Wilcoxon rank-sum test. R version 4.3.1 (R Foundation for Statistical Computing, Vienna, Austria) was used to perform all statistical analyses. Statistical significance was set at a *p* value of < 0.05.

### Study 1: risk factor analysis for the development of bacterial pneumonia among patients with esophageal motility disorders

We compared the baseline characteristics between patients with EMDs who developed BP 1 year before diagnosis and others using univariate analysis to identify risk factors for the development of BP among patients with EMDs. Factors showing a *p* value of < 0.05 from univariate analysis were selected as candidate factors to be fit in multivariable analysis. Factors adjusted for each candidate factor in multivariable analysis were selected from baseline characteristics using a directed acyclic graph (DAG) to identify confounding factors based on causal association (Supplementary Fig. 1) [18, 19]. Multivariable logistic regression models were used to estimate the odds ratios (ORs) and 95% confidence intervals (CIs) of the candidate factors. Data of missing values of the candidate factors were analyzed using multiple imputations (number of multiple imputations = 5, number of interactions = 1000) by chained equations using baseline variables (age, sex, BMI, duration of symptom, previous invasive treatment, ASA-PS, ECOG-PS, pretreatment Eckardt score, manometric diagnosis, IRP, dilation grade, food retention, and morphology), auxiliary variables (length of myotomy and procedure time of POEM), and the outcome (BP) (R packages: “mice,” “norm2,” and “miceadds”).

### Study 2: effect of peroral endoscopic myotomy for developing bacterial pneumonia among patients with esophageal motility disorders

Time-varying covariates occurred when the status of the covariates changed during the follow-up period. Considering treatment status (either “never POEM” or “ever POEM”) to be a time-varying covariate, the effect of treatment status for the development of BP was estimated by the Kaplan–Meier method [20, 21], and its significance was evaluated by the Cox proportional hazard model (R packages: “survival,” “survminer,” “rms,” “ggplot2”). Regarding the follow-up time among the patients who underwent POEM, misclassifying the interval from disease onset to the date of POEM administration as the period in the treatment status of “ever POEM” or intentionally excluding the interval from the analysis would induce misclassification bias or selection bias, respectively (Supplementary Fig. 2). Therefore, the total follow-up time was split into short intervals according to both the treatment status and the event occurrence; data from multiple observations per individual were combined to calculate the significance of the Cox proportional hazard model using “cluster” augment in the “coxph” function of R package “survival” [22].

## Results

### Patient selection process and baseline characteristics

**Fig. 1:**
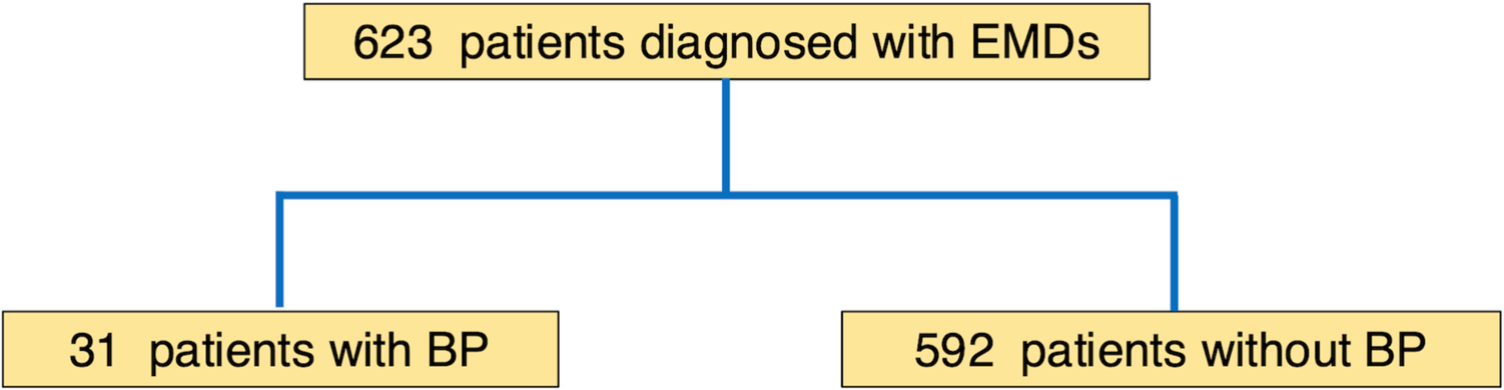
Flowchart of patients with or without bacterial pneumonia within 1 year before diagnosis of esophageal mortality disorders. *BP* Bacterial pneumonia, *EMDs* Esophageal mortality disorders

A flow diagram of the patient selection process is shown in Fig. 1. A total of 623 patients diagnosed with achalasia and non-achalasia were enrolled. Patient characteristics are summarized in Table 1. The median patient age was 52 years (IQR, 40 – 69 years). The median BMI was 21.0 kg/m^2^ (IQR, 18.9– 23.5 kg/m^2^). A total of 486 patients (77.8%) had achalasia according to the Chicago classification v3.0 by HRM, including 338 (54.3%) with type I, 108 (17.3%) with type II, and 40 (6.4%) with type III achalasia. After excluding patients with achalasia, 25 (4.0%) patients had DES, 15 (2.4%) had jackhammer esophagus, and 15 (2.4%) had EGJOO. The remaining nine patients (1.4%) were also included in the study. Morphology revealed that 156 patients (25.1%) had sigmoid esophagus, with dilatations of 1 in 258 patients (41.4%), 2 in 335 (53.8%), and 3 in 23 (3.7%). Food retention was observed during endoscopy in 215 patients (34.5%).

**Table 1:** Baseline characteristics of patients^a^

	*n* = 623
Age at diagnosis, median (IQR), years	52 (40–69)
Sex (male), *n* (%)	306 (49.1)
BMI, median (IQR), kg/m^2^	21.0 (18.9–23.5)
Duration of symptom, median (IQR), years	4.4 (2.0–10.5)
Missing, *n* (%)	1 (0.16)
Previous invasive treatment, *n* (%)	150 (24.1)
Pneumatic dilation, *n* (%)	126 (20.2)
Heller myotomy, n (%)	17 (2.7)
POEM, *n* (%)	7 (11.2)
ASA-PS, *n* (%)	
1	480 (77.0)
2	123(19.7)
3	18 (2.9)
4	2 (0.32)
ECOG-PS, *n* (%)	
0	527 (84.6)
1	80 (12.8)
2	8 (1.3)
3	8 (1.3)
Pretreatment Eckardt score, median (IQR)	5 (4–7)
Dysphagia	3 (3–3)
Regurgitation	1 (1–2)
Weight loss	0 (0–1)
Chest pain	1 (0–1)
Missing, *n* (%)	5 (0.8)
Chicago classification	
Achalasia, *n* (%)	486 (77.8)
Type 1	338 (54.3)
Type 2	108 (17.3)
Type 3	40 (6.4)
Achalasia variants, *n* (%)	
Distal esophageal spasm	25 (4.0)
Jackhammer esophagus	15 (2.4)
EGJ outflow obstruction	15 (2.4)
Others	9 (1.4)
Missing, *n* (%)	73 (11.7)
IRP, median (IQR), mmHg	28.3 (19.4–38.0)
Missing, *n* (%)	78 (12.5)
Morphology	
Straight type, *n* (%)	464 (74.5)
Sigmoid type 1, *n* (%)	112 (18.0)
Sigmoid type 2, *n* (%)	44 (7.1)
Missing, *n* (%)	3 (0.48)
Dilation grade	
Grade 1, *n* (%)	258 (41.4)
Grade 2, *n* (%)	335 (53.8)
Grade 3, *n* (%)	23 (3.7)
Missing, *n* (%)	7 (1.1)
Food retention, *n* (%)	215 (34.5)
Missing, *n* (%)	4 (0.64)

^a^*POEM* Peroral endoscopic myotomy, *IQR* Interquartile range, *BMI* Body mass index, *ASA-PS* American Society of Anesthesiologists-physical status, *ECOG-PS* Eastern Cooperative Oncology Group-physical status, *IRP* Integrated relaxation pressure, *EGJ* Esophagogastric junction

### Comparison of baseline characteristics between esophageal motility disorder patients with and without bacterial pneumonia

Of the 623 patients, 31 (5.0%) had BP within 1 year before the diagnosis of EMDs. Table 2 presents the results of the univariate analysis comparing the baseline characteristics between patients in the EMDs with and without BP within 1 year before the diagnosis of EMDs. Patients in the EMDs with BP group were older (*p* = 0.021), had a lower BMI (*p* = 0.0070), lower IRP (*p* = 0.012), and more manometric diagnoses of spastic esophageal disorders (*p* = 0.019) than those in the EMDs without BP group.

**Table 2:** Comparison of baseline characteristics between esophageal motility disorders patients with and without bacterial pneumonia^a^

	EMDs with BP *n* = 31	EMDs without BP *n* = 592	*p* value
Age, median (IQR), years	61 (48.0–77.5)	52 (40.0–69.0)	0.021^*^
Sex (male), *n* (%)	21 (67.7)	285 (48.1)	0.052
BMI, median (IQR), kg/m^2^	19.3 (16.8–21.1)	21.1 (19.0–23.5)	0.0070^*^
Duration of symptom, median (IQR), years	5.9 (1.6–10.3)	4.4 (2.0–10.5)	0.88
Previous invasive treatment, *n* (%)	8 (25.8)	142 (24.0)	0.99
ASA-PS > 1, n (%)	8 (25.8)	130 (22.0)	0.72
ECOG-PS ≥ 1, *n* (%)	8 (25.8)	88 (14.9)	0.12
Pretreatment Eckardt score, median (IQR), points	5 (4.0–6.5)	5 (4.0–7.0)	0.31
IRP, median, median (IQR), mmHg	18.9 (15.2–29.2)	28.5 (19.9–38.2)	0.012^*^
Manometric diagnosis (spastic esophageal disorders), *n* (%)	8 (25.8)	72 (12.2)	0.019^*^
Dilation grade 1, *n* (%)	13 (41.9)	245 (41.4)	1.0
Food retention, *n* (%)	13 (41.9)	202 (34.1)	0.50
Morphology (sigmoid type), *n* (%)	7 (22.6)	149 (25.2)	0.91

^a^*BP* Bacterial pneumonia, *EMDs* Esophageal motility disorders, *IQR* Interquartile range, *BMI* Body mass index, *ASA-PS* American Society of Anesthesiologists-physical status, *ECOG-PS* Eastern Cooperative Oncology Group-physical status, *IRP* Integrated relaxation pressure

^*^p < 0.05

### Causal association with bacterial pneumonia among patients with esophageal motility disorders

We performed multivariable logistic regression analysis for each variable using the following four variables based on DAG: age at diagnosis, BMI, IRP, and manometric diagnosis of spastic esophageal disorders. In addition, the percentage of missing values across the 12 variables varied from 0% to 11.7%. Variables most associated with missing data were the Chicago classification and IRP. We believe that most of these cases were caused by esophageal flexion or strong contraction of the esophagogastric junction, which prevented the catheter from passing into the stomach, resulting in some patients not being able to tolerate the insertion of a catheter into their nose. We considered that most of the data were missing not at random and used multiple imputation (MI) to handle the missing data and create and analyze five multiply imputed datasets using multivariate analysis. A complete case analysis was performed for the sensitivity analysis of MI. Table 3 lists the results of the multivariate analysis in which MI was performed. Older age (OR = 1.29, 95% CI 1.04–1.59, *p* = 0.019; 10-year increments), lower BMI (OR = 0.87, 95% CI 0.78–0.98, *p* = 0.026), and manometric diagnosis of spastic esophageal disorders (OR = 2.97, 95% CI 1.24–7.16, *p* = 0.015) were independent risk factors associated with development of BP. The results of the multivariate analysis performed with complete analysis were not significantly different from those of the multivariate analysis with MI.

**Table 3:** Causal association with bacterial pneumonia among patients with esophageal motility disorders^a^

	Multiple imputation analysis			Complete case analysis		
	OR	95% CI	*p*-value	OR	95% CI	*p*-value
Age, 10-year increments	1.29	1.04–1.59	0.019^*^	1.29	1.05–1.61	0.019^*^
BMI, 1-kg/m^2^ increments^b^	0.87	0.78–0.98	0.026^*^	0.88	0.77–0.98	0.025^*^
IRP, 10-mmHg increments^c^	0.76	0.55–1.05	0.10	0.76	0.54–1.03	0.10
Manometric diagnosis (spastic esophageal disorders)	2.97	1.24–7.16	0.015^*^	2.97	1.18–6.95	0.015^*^

^a^*OR* Odds ratio, *CI* Confidence interval, *BMI* Body mass index, *IRP* Integrated relaxation pressure

Variables were adjusted for ^b^age, pretreatment Eckardt score, and ^c^ manometric diagnosis

^*^p < 0.05

### Time-varying cox regression method

**Fig. 2:**
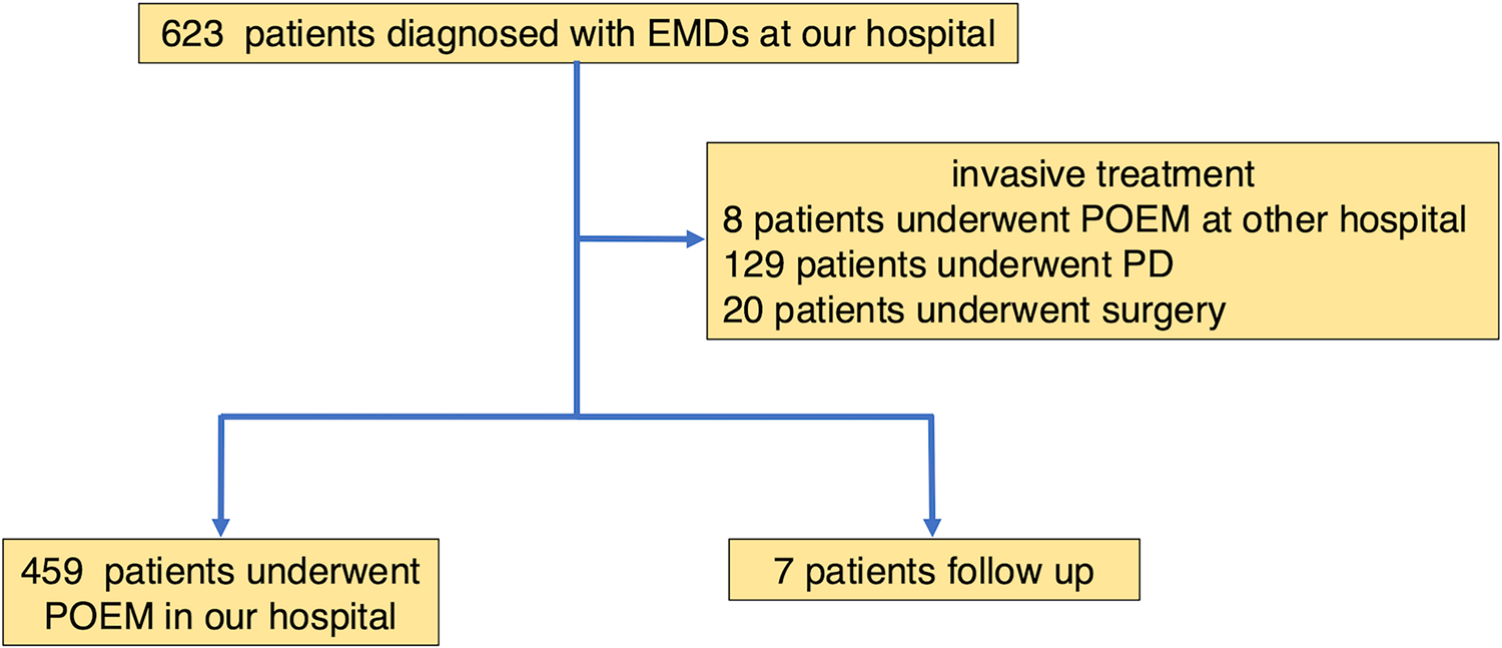
Flowchart of patients in time-varying method of analysis. *PD* Pneumatic dilation; *EMDs* Esophageal motility disorders, *POEM* Peroral endoscopic myotomy

**Fig. 3:**
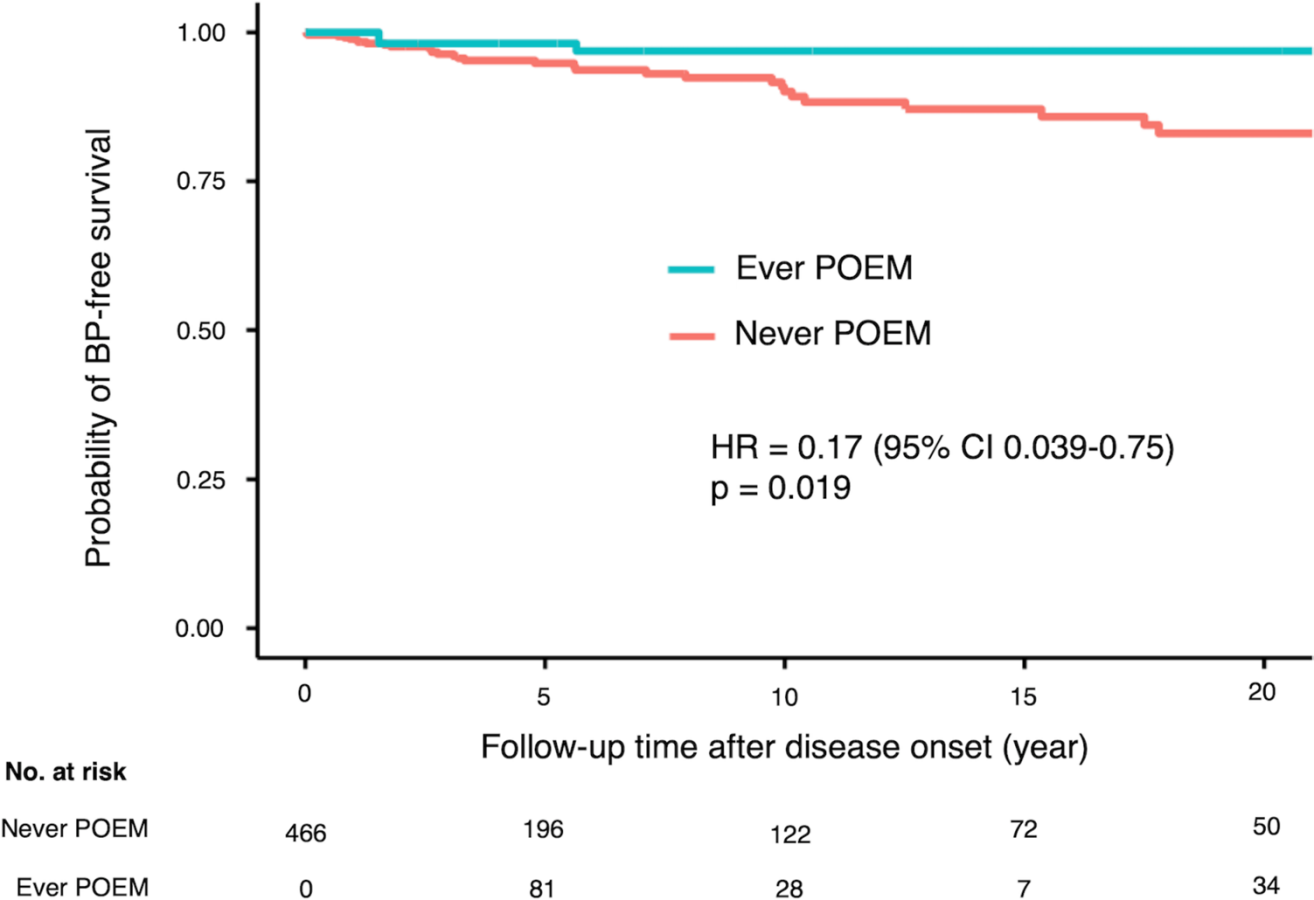
Kaplan–Meier curve of the probability of bacterial pneumonia-free survival stratified by treatment status. BP is significantly less frequent in the “Ever POEM” group than in the “Never POEM” group (hazard ratio = 0.17, 95% confidence interval: 0.039–0.75, *p* = 0.019). *BP* Bacterial pneumonia, *POEM* Peroral endoscopic myotomy, *HR* Hazard ratio

Of the original 623 patients, eight who underwent POEM at other hospitals, 129 who underwent PD, and 20 who underwent surgery were excluded, resulting in the inclusion of 466 patients in the study (Fig. 2). Of these, 459 underwent POEM in our hospital and seven were followed up with. Figure 3 shows the Kaplan–Meier curve adjusted for the time of POEM (time-varying method of analysis). Cox proportional hazards regression performed using the time-varying method of analysis [20], with adjustment for age and manometric diagnosis of spastic esophageal disorders, revealed that the frequency of BP in the group that underwent POEM was significantly lower (HR = 0.17, 95% CI 0.039–0.75, *p* = 0.019). Patient age at the beginning of the observation period was used as an adjustment variable.

## Discussion

This study enrolling 623 patients diagnosed with EMDs at our hospital revealed that the risk factors for developing BP in patients with EMDs were older age, lower BMI, and a manometric diagnosis of spastic esophageal disorder. Patients with EMDs, such as those with esophageal achalasia and non-achalasia, sometimes develop BP and pyothorax, and individual cases have been previously reported [3–6]. This is the first report to identify the risk factors for BP in patients with EMDs. This study also showed that POEM might help prevent BP development by improving risk factors for BP development and reducing respiratory complications such as atelectasis and aspiration observed in patients with EMDs.

BP is pneumonia caused by bacterial pathogens; it is an infectious disease that develops when pathogens reach the upper or lower respiratory tract and their virulence overcomes host defenses [7, 13]. A variety of risk factors are known to contribute to the development of pneumonia, with a decline in host immune function and factors known to predispose to aspiration, such as old age, impaired swallowing, decreased consciousness, increased chance of esophageal contents reaching the lungs, and dentition [7]. BP is a pneumonia caused by bacterial pathogens; however, despite improvements in diagnostic techniques, the identification rate of pneumonia-causing organisms based on sputum culture testing has been reported to be as low as 40–50% [14]. In addition, in up to 77% of pneumonia cases in older adults, the causative pathogen has not been identified. Therefore, due to the low identification rate of causative bacteria, in this study, we defined BP occurring in patients with EMDs as pneumonia requiring antibiotic treatment.

Cases of pneumonia and pyothorax have long been reported in patients with esophageal peristalsis disorders such as achalasia [3–6]. Furthermore, a significantly higher incidence of aspiration pneumonia has been reported in patients with achalasia [23]. Structural and functional changes throughout the respiratory system are said to lead to a reduction in host defense capacity [13]. Previous reports have demonstrated that approximately half of patients with achalasia have structural changes in the airways and lung parenchyma due to repetitive aspiration [24, 25]. Such structural lung abnormalities, including atelectasis [26], may lead to decreased airway clearance and contribute to BP development [27, 28].

Our study’s results showed that the prevalence of BP in 623 patients diagnosed with EMDs at our hospital was 5.0%. In an observational study in Japan in which 259 out of 9,930 older patients (2.6%) in geriatric and long-term care facilities developed pneumonia due to aspiration within 3 months [29]. Our result was higher than this reported prevalence of pneumonia due to aspiration in the elderly population who have risk of developing pneumonia; however, the median age of the patients in this study was 52 years (IQR, 40–69 years), suggesting that patients with EMDs may have a higher incidence of BP due to aspiration than may those without EMDs as in previous reports [23]. In a study reporting the prevalence of respiratory symptoms in 110 patients diagnosed with achalasia [30], aspiration (sensation of inhaling regurgitated esophagogastric material) was found in 31 patients (34%), which was higher than the percentage of patients who developed BP in our study. The reason for the difference in these results is that the outcome of our study was not aspiration but the development of BP. Therefore, if a similar definition of aspiration had been used in our study, the results might have been comparable to previously reported results.

The present study analyzed the risk factors associated with the development of BP in patients with EMDs and showed that older age, lower BMI, and a manometric diagnosis of spastic esophageal disorders were risk factors. Older age is a known risk factor for the development of BP [13]. In this study, older age was identified as a risk factor for BP in patients with EMDs, which may be due to a decline in swallowing function and poor immune function associated with old age [7]. Furthermore, this study’s results indicate that a lower BMI is also a risk factor for developing BP, which may possibly be related to sarcopenia. Sarcopenia due to malnutrition is a known risk factor for pneumonia because it causes skeletal muscle loss and is associated with impaired immune function, dysphagia, and respiratory muscle weakness [31, 32]. BMI is widely used as a measure of nutritional status [33], and a lower BMI (i.e., lower nutrition) may result in poor immune function, dysphagia, and respiratory weakness due to sarcopenia, leading to the development of BP [32]. Moreover, in this study, we found that manometric diagnosis of spastic esophageal disorder is a risk factor for the development of BP in patients with EMDs. We initially considered that the long duration of symptoms might cause esophageal dilation and tortuosity, leading to food retention in the esophagus, which might cause BP due to aspiration. However, the results of this study showed that neither duration of symptoms, esophageal dilatation and sigmoid esophagus, nor endoscopic food retention were risk factors for the development of BP in patients with EMDs. Regarding food retention, we assessed the causal association based on DAG (Supplementary Fig. 1), with age and Pre Eckardt score as confounding variables adjusted to avoid bias to underestimate or overestimate the causal relationship between food retention and BP. However, no clear causal association was found. Therefore, we hypothesized that the spasm itself was closely associated with the development of BP. Carlson et al. used a functional lumen imaging probe to demonstrate that repetitive retrograde contractions could be observed in patients with type 3 achalasia [34]. In addition, Park et al. demonstrated luminal closure during the early period of peristalsis and subsequent bolus segmentation by evaluating the bolus flow patterns of spasm in a similar cohort [35]. These reports suggest that, even in the absence of food retention, the spasm in the esophagus itself is likely to induce bolus reflux, resulting in aspiration and BP.

The results of the time-varying Cox regression analysis suggest that performing POEM may reduce the risk of developing BP in patients with EMDs. We suggest that this may take place through several mechanisms. First, there is improvement in bolus reflux and dysphagia by improving esophageal spasm and esophageal transit [36]. These improvements may require adequate lower esophageal sphincter myotomy for achalasia, including spasm of the esophageal body, for spastic esophageal disorders [37]. Second, POEM ameliorates malnutrition, which is a risk factor for BP development in patients with EMDs. We have reported that performing POEM in preoperatively malnourished patients with EMDs increases skeletal muscle mass after POEM [38]. Skeletal muscle mass increases after POEM, which may result in decreased dysphagia and risk of developing BP. Third, POEM may improve tracheal compression by the dilated esophagus and food aspiration, improve structural and functional changes throughout the respiratory system, such as atelectasis, and reduce the incidence of BP in patients with EMDs by improving airway clearance [24–28]. As mentioned above, although performing POEM may contribute to a reduction in the incidence of BP, it appears that this procedure may not directly prevent BP, rather it may indirectly contribute to a reduction in the incidence of BP by alleviating the risk factors for BP development and reducing respiratory complications seen in patients with EMDs. To examine whether POEM can prevent the development of BP, we excluded patients who underwent invasive treatments other than POEM, such as pneumatic dilation and surgery, in Study 2. Therefore, we did not examine whether treatment other than POEM can prevent of the developing BP in this study. However, we believe that POEM is not the only treatment that reduces the risk of developing BP; to prevent the development of BP in patients with EMDs, it is important to provide effective treatment for patients with EMDs. Therefore, even if pneumatic dilation and surgery are undergone instead of POEM, the patient’s risk of developing BP may be reduced if the treatment is effective.

Based on our study’s results, older patients, those with a lower BMI, and those with a manometric diagnosis of spastic esophageal disorders would benefit from undergoing POEM to prevent the development of BP. However, care should be taken when deciding whether POEM should be performed to prevent BP development. POEM in older patients has been reported to be equally effective and safe, equivalent to that for non-elderly patients in studies by Tang et al. [39]; however, these were retrospective studies that included a small number of patients. In addition, older individuals, who have a lower physiological reserve and a greater burden of comorbidities, are expected to have a lower tolerance for adverse events [40]. POEM in patients aged > 80 years is associated with significantly higher rates of adverse events not related to the procedure [41]. Moreover, malnutrition is also associated with adverse perioperative events [42]. Concerning spastic esophageal disorder, achalasia variants (including spastic esophageal disorders) are potential barriers for POEM treatment [43]. Therefore, considering the preventive potential of POEM for BP, the decision to perform it in patients with any of the aforementioned risk factors requires a careful balancing between the advantages of preventing BP and adverse events that may result from the treatment. The present study reveals that POEM may prevent the onset of BP, however, when is the best time to use POEM to prevent the onset of BP? In study 2, many of the patients in our survival analysis developed BP early after disease onset, and most developed BP within 5 years.

Therefore, POEM is recommended as early as possible in patients affected by EMDs to prevent the development of BP.

This study has some limitations. First, this was a single-center, retrospective study with selection, recall, and information biases. However, randomized controlled trials cannot accurately evaluate the effect of preventing BP because it cannot consider the time between the onset and diagnosis of EMDs. Therefore, we had to choose this study design. Second, in the Cox regression analysis considering time-varying covariates, the hazard ratio of treatment status was adjusted by the manometric diagnosis of spastic esophageal disorders, based on the assumption that manometric diagnosis in our institution was maintained throughout the clinical course. Third, in this study of Japanese patients with EMDs, a lower BMI was identified as a risk factor for developing BP. However, since most patients with EMDs from Western populations have a normal or high BMI [44], this result may not necessarily apply to them. Finally, we could not adjust for BMI, which was proven to be a significant factor associated with the development of BP in the Cox regression analysis, because BMI had not been recorded throughout the patients’ clinical courses.

In conclusion, the risk factors associated with the development of BP in patients with EMDs were older age, lower BMI, and manometric diagnosis of spastic esophageal disorders. In patients with EMDs, POEM could decrease spasm-related bolus reflux and improve patients’ nutritional status through resolution of transit disturbance, and reduce the development of respiratory complications. It is suggested that POEM might help prevent BP development by improving esophageal motility disorders and undernutrition, which are considered risks for BP development, and by reducing the incidence of respiratory complications.

## Supplementary Information

Below is the link to the electronic supplementary material.

Supplementary file1 Fig. S1 Directed acyclic graph for finding confounders based on causal association. The outcome was bacterial pneumonia; blue circles indicate observed variables and gray circles indicate unobserved variables. Factors adjusted for each candidate factor in the multivariate analysis were selected using the DAG. BMI: body mass index, ASA-PS: American Society of Anesthesiologists-physical status, ECOG-PS: Eastern Cooperative Oncology Group-physical status, IRP: integrated relaxation pressure, DAG: directed acyclic graph (TIFF 6902 KB)

Supplementary file2 Fig. S2 Explanation of the time-varying method (A) Misclassification bias: In patients who underwent POEM during the follow-up period, misclassification of the interval time, which was the time from disease onset to the date of POEM, to the period in the treatment status of “ever POEM” would result in misclassification bias. (B) Selection bias: In patients who underwent POEM during the follow-up period, exclusion of the interval time from the analysis intentionally introduced selection bias. (C) Time varying methods: In patients who underwent POEM during the follow-up period, the interval time (the period of time between disease onset to the date of POEM) was classified as the “Never POEM” treatment status period, which resulted in an analysis that considered time-varying covariates. POEM: peroral endoscopic myotomy (TIFF 6902 KB)
