# Supplementary figures and images for: Factors associated with the development of bacterial pneumonia and the preventive potential of peroral endoscopic myotomy in patients with esophageal motility disorders: a case–control study
Source: J Gastroenterol. 2025 Mar 24;60(8):947–55. doi: 10.1007/s00535-025-02238-8 (PMC12289750; doi:10.1007/s00535-025-02238-8)

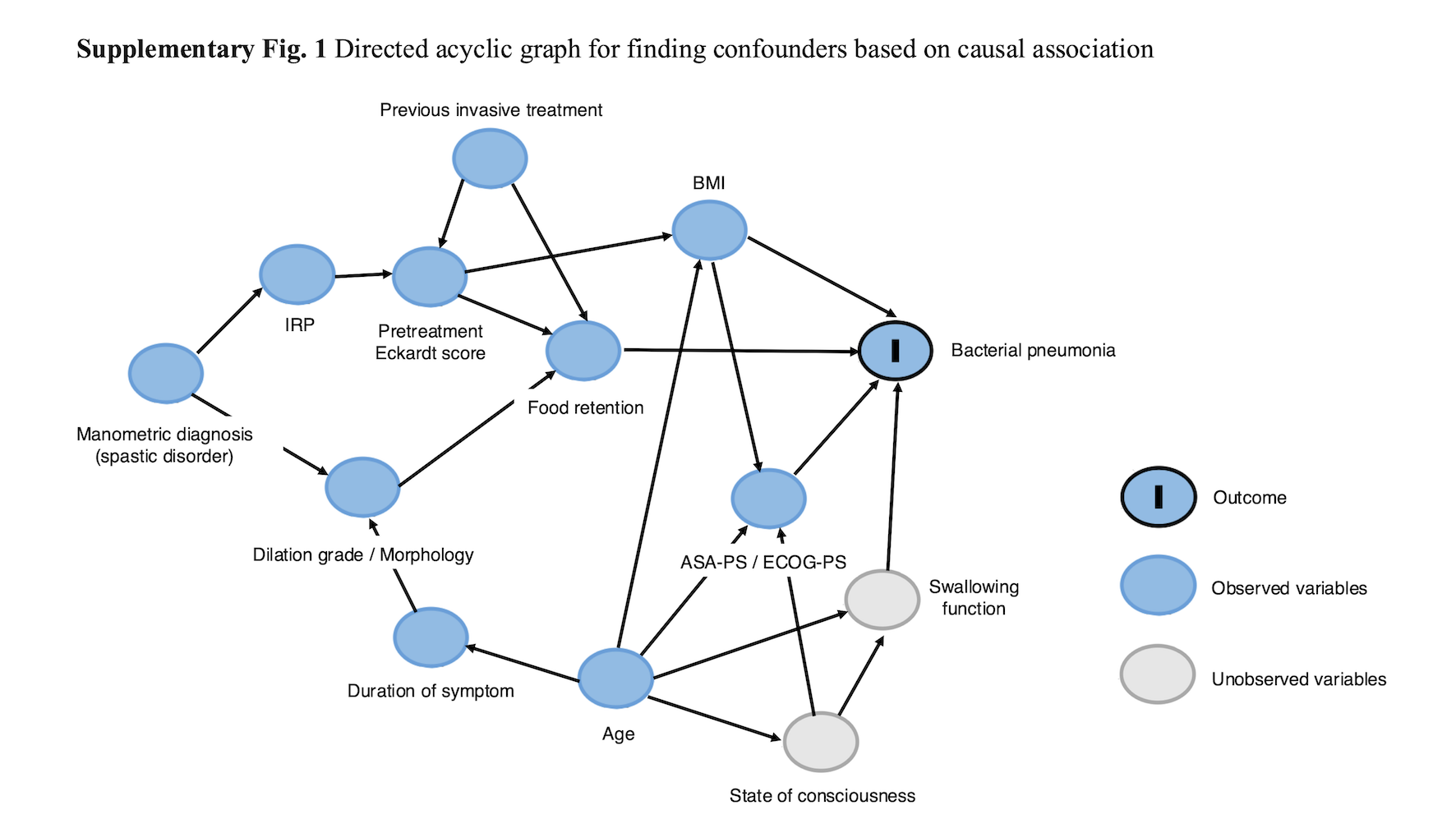

Supplement: Supplementary file 1 — Supplementary file1 Fig. S1 Directed acyclic graph for finding confounders based on causal association. The outcome was bacterial pneumonia; blue circles indicate observed variables and gray circles indicate unobserved variables. Factors adjusted for each candidate factor in the multivariate analysis were selected using the DAG. BMI: body mass index, ASA-PS: American Society of Anesthesiologists-physical status, ECOG-PS: Eastern Cooperative Oncology Group-physical status, IRP: integrated relaxation pressure, DAG: directed acyclic graph (TIFF 6902 KB) [file 535_2025_2238_MOESM1_ESM.tiff]

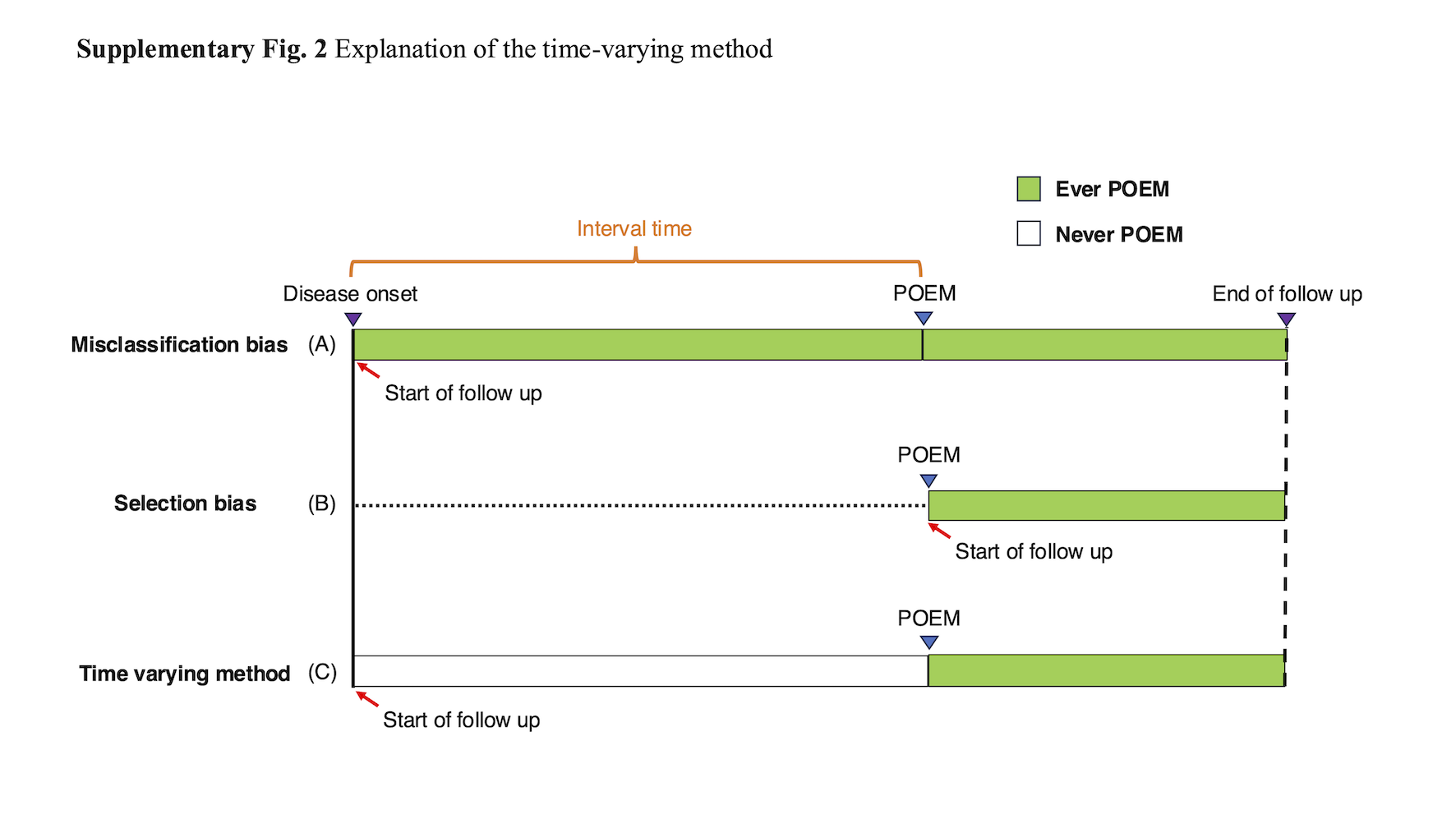

Supplement: Supplementary file 2 — Supplementary file2 Fig. S2 Explanation of the time-varying method (A) Misclassification bias: In patients who underwent POEM during the follow-up period, misclassification of the interval time, which was the time from disease onset to the date of POEM, to the period in the treatment status of “ever POEM” would result in misclassification bias. (B) Selection bias: In patients who underwent POEM during the follow-up period, exclusion of the interval time from the analysis intentionally introduced selection bias. (C) Time varying methods: In patients who underwent POEM during the follow-up period, the interval time (the period of time between disease onset to the date of POEM) was classified as the “Never POEM” treatment status period, which resulted in an analysis that considered time-varying covariates. POEM: peroral endoscopic myotomy (TIFF 6902 KB) [file 535_2025_2238_MOESM2_ESM.tiff]
